# Heroin detection in a droplet hosted in a 3D printed support at the miniaturized electrified liquid-liquid interface

**DOI:** 10.1038/s41598-022-21689-0

**Published:** 2022-11-03

**Authors:** Paulina Borgul, Karolina Sobczak, Karolina Sipa, Konrad Rudnicki, Slawomira Skrzypek, Anna Trynda, Lukasz Poltorak

**Affiliations:** 1 Electroanalysis and Electrochemistry Group, Department of Inorganic and Analytical Chemistry, Faculty of Chemistry, University of Lodz, Tamka 12, 91-403 Lodz, Poland; 2 Chemistry Department, Central Forensic Laboratory of the Police, Warsaw, Poland

**Keywords:** Analytical chemistry, Electrochemistry

## Abstract

Simple sensing protocols for the detection of illicit drugs are needed. Electrochemical sensing is especially attractive in this respect, as its cost together with the analytical accuracy aspires to replace still frequently used colorimetric tests. In this work, we have shown that the interfacial transfer of protonated heroin can be followed at the electrified water-1,2-dichloroethane interface. We have comprehensively studied the interfacial behavior of heroin alone and in the presence of its major and abundant cutting agents, caffeine and paracetamol. To maximally increase developed sensing protocol applicability we have designed and 3D printed a platform requiring only a few microliters of the aqueous and the organic phase. The proposed sensing platform was equipped with a cavity hosting a short section of Ag/AgCl electrode, up to 20 µL of the aqueous phase and the end of the micropipette tip being used as a casing of a fused silica capillary having 25 µm as the internal pore diameter. The volume of the organic phase was equal to around 5 µL and was present inside the micropipette tip. We have shown that under optimized conditions heroin can be detected in the presence of caffeine and paracetamol existing in a sample with 10,000 times excess over the analyte of interest. The calculated limit of detection equal to 1.3 µM, linear dynamic range spanning to at least 50 µM, good reproducibility, and very low volume of needed sample is fully in line with forensic demands.

## Introduction

As many as 62 million people were using opioids for non-medical purposes in 2019-estimation says^1^ Opioids are especially prevalent in North America. In Europe, although very popular, make a smaller contribution to the total illicit drugs market. Heroin is the best known and most common opiate. Only in 2019, heroin trafficking was confirmed in 99 countries. In 2019 the amount of seized heroin in Europe was record-breaking and has tripled since 2016. For the given year, heroin represented 27% of the total number of drugs sold worldwide.

Heroin (systematic name (IUPAC): (5α, 6α)-7,8-didehydro-4,5-epoxy-17-methylmorphinan-3,6-diol acetate) is also known as diacetylmorphine and diamorphine. It can be synthesized by acetylation of morphine or extracted from poppy seeds where it occurs naturally. Heroin is classified as a drug with the highest addiction potential^2^. In a pure form it appears as a white powder^3^, which is rare for the street samples^4,5^. The color of the illegal heroin samples vary depending on their contamination originating from the production process, added cutting agents, and ranges from white, through cream, beige, to medium and dark brown (almost black)^6^. In the European Union, heroin purity at the retail level is estimated within the range from 18 to 30% occasionally being elevated^7^. The street sample filling is mainly assured with cutting agents which are commonly added to many illicit drugs and make an additional burden to the sensing device's functionality^8–11^. The main cutting agents of heroin are paracetamol and/or caffeine, which were found in 90% (!) of heroin street samples in Europe. According to an elegant review by Broséus et al. the presence of these adulterants in seized heroin powders was confirmed in 128 countries^12^. Accordingly, there is an obvious need for a sensing methodology development that can detect heroin directly from street samples. At the same time, illegal sample testing is expected to be selective, simple in terms of manufacturing, portable, easy in operation, and cheap. In a view of heroin street samples composition, the sensing device providing linear dynamic range within µM range will suffice. These requirements are within the capability of electrochemical detection. This broadly defined methodology appears to be an excellent choice for the rapid and highly sensitive determination of narcotic substances^13^. Drugs such as cocaine^14,15^, fentanyl^16,17^, or tetrahydrocannabinol^18,19^ can be directly detected by following their consecutive redox reactions or indirectly after smart electrode surface engineering^20^. A few electroanalytical works focused on the analyte of interest exist in the literature. Rodriguez et al*.* studied direct oxidation of heroin at carbon paste electrodes providing similar output as compared with the high-performance liquid chromatography^21^. Garrido et al*.* investigated the voltammetric oxidation of heroin and its metabolites on glassy carbon electrodes and proposed the mechanism of its oxidation^22^. The strategy of electrochemically detecting heroin in street samples was comprehensively studied by Florea et al*.* with graphite screen-printed electrode with focus given to two pH values, this is 7.0 and 12.0^23^.

With tertiary amine functionality present within heroin structure, this illicit drug may be protonated at pH < pK_a_ (7.6)^24^, and hence, positively charged. As such, heroin may undergo electrochemically controlled interfacial ion transfer reaction across the electrified liquid–liquid interface (eLLI) which was confirmed in a work published by Gulaboski et al. (the referred study was focused on several opioids, amphetamine-like drugs, their metabolites and partition coefficients calculations)^25^. Electrochemical studies at the eLLI not only provide direct insight into molecular partitioning and pharmacochemical parameters but also can be directly employed in the detection based on the simple interfacial ion transfer reactions (sensing not limited to oxidation/reduction reactions)^26^. In a view of illicit drugs sensing, eLLI was successfully applied to detect and fully understand the interfacial behavior of cocaine together with its numerous cutting agents^8,27^. Also, we have shown that ionic currents originating from the ephedrine (amphetamine/methamphetamine precursor, doping agent in sport) transfer across eLLI suffice for its quantification in urine^28,29^. Reports describing the interfacial behavior of other regulated molecules such as gamma-aminobutyric acid^30^, and a few opioids and amphetamine-like drugs^25^ are also available in the literature.

Our work is focused on understanding the heroin behavior at the eLLI. The electroanalytical signals originating from the charged heroin species transfer across the polarized soft junction were used to plot ionic partition diagrams (providing several pharmacochemical parameters), derive physicochemical, and analytical descriptors (LOD, LOQ, voltammetric detection sensitivity). Heroin sensing was performed in the presence of its major cutting agents: caffeine and paracetamol. As reported by Kaliszczak et al. caffeine is active at the electrified water-1,2-dichloroethane interface, and hence, may potentially interfere during heroine detection^31^. We have proposed a very straightforward sensing platform that is derived from a 3D printed support with a cavity hosting a few microliters of the aqueous phase (heroin was always initially present in this compartment) and a single pore microcapillary being filled with a few µL of the organic phase. eLLI miniaturization has a few crucial benefits over traditional macroscopic counterparts: (i) minimal consumption of the chemicals; (ii) lower capacitive currents; (iii) higher mass transfer to eLLI and (iv) improved stability of the soft junction governed by the pre-adjusted microscopic aperture surface wettability^32^. Approaches used to miniaturize eLLI can be based on metal wire templated glass capillary^33^, pulled glass capillaries^30,34^, patterned glass slides^35^, laser-ablated polymeric films^36^, micro-punched films^28,37^, apertures fabricated in the SiN chips^38–40^, or simply are based on porous materials such as zeolites, mesoporous silica of fiberglass membranes^41–44^. Miniaturization protocols are also frequently applied during sample preparation aiming at e.g. analyte extraction from the investigated matrix^45^. In this work, the eLLI was miniaturized with the fused silica microcapillary hosted in a micropipette tip^46^. Heroin, caffeine and paracetamol formulations (model street samples) were subjected to analysis in the constructed sensing platforms using only a few µL of each phase.

## Experimental section

### Materials

Heroin (M = 369.42 g·mol^−1^, Cayman Chemical Company); paracetamol (M = 151.16 g·mol^−1^, Synoptis Pharma tablets); caffeine (M = 194.19 g·mol^−1^, Argenta); tetrapropylammonium chloride (M = 221.81 g·mol^−1^, Alfa Aesar), sodium chloride (M = 58.44 g·mol^−1^, Fisher Chemical); 1,2 – dichloroethane (M = 98.96 g·mol^−1^, POCH); were all used as received. As the organic phase electrolyte, we have used a salt synthesized from the potassium tetrakis(4-chlorophenyl) borate (KTPBCl, M = 496.11 g·mol^−1^, Sigma–Aldrich) and bis(triphenylphosphoranylidene)ammonium chloride (BTPPACl, M = 574.03 g·mol^−1^, Sigma-Aldrich). KTPBCl and BTPPACl were first separately dissolved in the 1:2 MeOH:H_2_O mixture and further added dropwise to each other. Resulting precipitate (BTPPATPBCl) was recrystallized from acetone. Next, the appropriate mass of crystals was further dissolved in 1,2 – dichloroethane to obtain 5 mM solution serving as the organic phase. The aqueous phase used to form ITIES was either 10 mM NaCl or 10 mM NaCl in a Britton–Robinson Buffer (BRB) buffer with a pH in the range from 2 to 11 (most often pH 2 or 5.5). The value of pH was adjusted with 1 M NaOH. BRB buffer was prepared from a stock solution of the buffer matrix being a mixture of boric acid (H_3_BO_3_), ortho phosphoric acid (H_3_PO_4_), acetic acid (CH_3_COOH) and sodium chloride (NaCl). The desired pH value for BRB was adjusted with a pH meter (Orion STAR, A111, The Netherlands) using a polymer pH electrode (Polilyte Lab, Hamilton, Switzerland).

### Electrochemical measurements

All electrochemical experiments were performed with AUTOLAB 302 N potentiostat–galvanostat (Metrohm Autolab B.V., The Netherlands) supported with NOVA 1.11.1 software. The measuring system was studied with four electrode configuration. The macroscopic electrified liquid–liquid interface was polarized using two counter electrodes (Pt wires) placed in the aqueous and the organic phase. The potential drop across planar soft junction was measured between two Ag/AgCl electrodes each placed in the Luggin capillary present on the aqueous and the organic phase side of the liquid–liquid interface.

The liquid–liquid interface formed at the end of the tip located within the 3D printed microliter volume aqueous phase droplet was polarized using two electrode configuration. The Ag/AgCl wire was protruding through the bottom of the droplet cavity whereas Ag/AgTPBCl was immersed inside the micropipette tip and remained in contact with the organic phase.

Macroscopic liquid–liquid interface scheme:

$$\left.Ag\right|\left.AgCl\right|\left.\begin{array}{l}10\;mM\ NaCl\ or\ 10\;mM\ NaCl+10\;mM \ BRB+\\ x \upmu M \ heroin; x \upmu M\ paracetamol;x \upmu M\ caffeine\end{array}\right|\left|\begin{array}{c}5\;mM \\ BTPPATPBCl\end{array}\right.\left|\begin{array}{c}10\;mM\ NaCl\\ 5\;mM\ BTPPACl\end{array}|AgCl\left|Ag\right.\right.$$

$$\text{Cell I}$$

Microscopic liquid–liquid interface scheme:

$$\left.Ag\right|\left.AgCl\right|\left.\begin{array}{c}10\;mM\ NaCl+10\;mM\ BRB+\\ x \upmu M\;heroin; x \upmu M\ paracetamol;x \upmu M\ caffeine\end{array}\right|\left|\begin{array}{c}5\;mM \\ BTPPATPBCl\end{array}\right.\left|AgTPBCl\left|Ag\right.\right.$$

$$\text{Cell II}$$

### 3D printing and sensing platform fabrication

3D printed platforms were designed in the Thinkercad software and further exported as the .stl files. All section of the constructed device were printed with either Prusa Mini + or Prusa i3 MK3S (Prusa, Czech Republic). Before fabrication, the design was sliced in the Prusa Slicer software using following parameters: 0.15 mm speed; PLA filament; 20% filling; 215 °C as the temperature for the heating nozzle for the first layer and 210 °C for the remaining layers; the table temperature was 60 °C during entire printing process. The polylactic acid filament was from Fibrelogy. The silver wire protruding through the aperture located below the cavity dedicated for the droplet deposition was secured with the silicone sealant to avoid any possible aqueous phase leakage. Before application for sensing, the silver wire was contacted to 1 M HCl and the sufficient anodic potential was applied against pseudoreference electrode (second Ag wire) so that the AgCl layer was formed at its surface.

The fused silica microcapillary based supports for the electrified liquid–liquid interface were prepared according to protocol published recently in our group^47^. First, the short section of the capillary having 25 µm as the pore internal diameter and hydrophobic pore interior was sliced with a ceramic knife (around 3–4 mm). Next, this section was inserted into the end of heat shrinkable micropipette tip dedicated for up to 20 µL volume. Bunsen burner was used to entrap the tip into shrined polymeric casing, whereas tweezers allowed the postiong of the capillary within the end of the tip so that only around 100 µm remained in contact with the tip. Again, we have used ceramic knife to remove the excess of the tubing. Microtip prepared in such manner was filled with the organic phase and was further inserted into the aqueous phase droplet using the 3D printed support.

## Results and discussion

**Figure 1:**
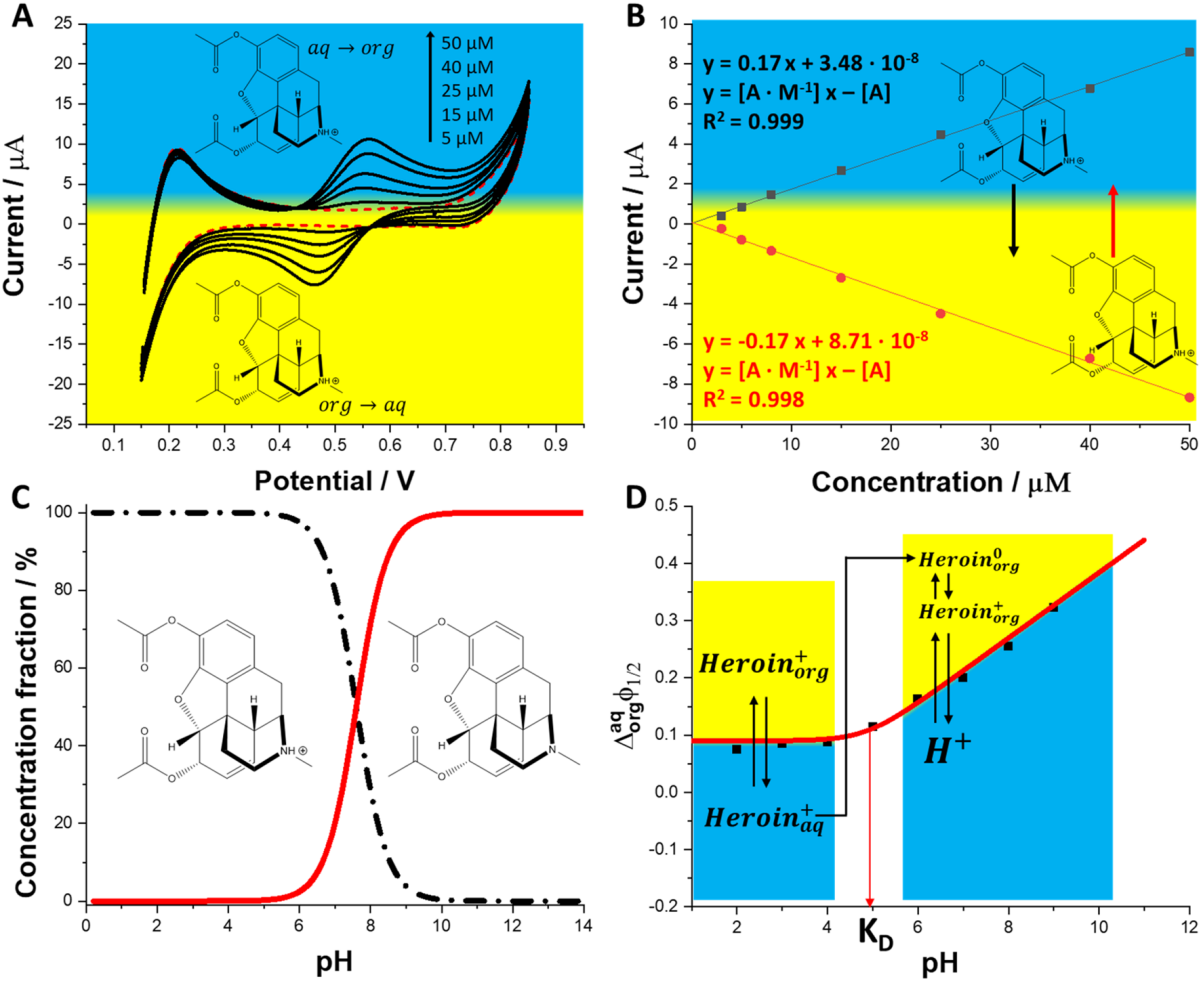
(**A**) – Cyclic voltammograms (CVs) recorded in the absence (red, dashed line) or in the presence (black, solid lines) of heroin initially present in the aqueous phase (10 mM HCl in 10 mM NaCl; pH = 2; scan rate 20 mV·s^−1^; concentrations of heroin are marked in the right upper corner of the figure). (**B**) – Calibration curves plotted based on the positive and negative ionic currents attributed to the heroin transfer from the aqueous to the organic and from the organic to the aqueous phase, respectively. Liner fitting equations are given next to the corresponding calibration curves. (**C**) – Concentration fraction diagram plotted for heroin (pK_a_ = 7.6). (**D**) – Ionic partition diagram plotted for heroin. For details see the corresponding discussion.

Below its pK_a_ value (7.6)^24^ tertiary amine present within heroin structure is protonated and hence, exists in a cationic form. It can transfer across the eLLI when sufficient Galvani potential difference is applied and the resulting ionic currents can be probed with all techniques offered by the electroanalytical toolbox. Figure 1A shows a series of cyclic voltammograms (CVs) recorded for the increasing concentration of the heroin initially present in the aqueous phase (10 mM HCl in 10 mM NaCl; pH = 2.0). At this pH and up to pH equal to around 6.0 (see Fig. 1C for the concentration fraction diagram) the fraction of a monocationic heroin is 100%. Positive peak current is due to heroin cation transfer from the aqueous to the organic phase whereas the negative signal is its back transfer. Simple ion transfer reaction is reversible as the positive to negative signal intensity ratio is equal to 1 (see the slope of the positive and negative calibration curves from Fig. 1B; Slope^+^ (0.17)/Slope^−^ (0.17) = 1), whereas the peak to peak separation is 73 mV for 15 µM heroin (the value being slightly higher than 59 mV expected for monocationic species and is due to the additional resistance originating from the organic phase). Based on the obtained CVs we have plotted the calibration curves (see Fig. 1B) ranging from 1 to 50 µM. Chosen concentration range is in line with the amount of heroin that can be found in street samples. Few mg of street powder containing from 10 to 30% of heroin dissolved in 1 mL of appropriate buffer and further diluted by a factor of 1000 falls within the studied linear dynamic range. The reported calibration curve (Fig. 1B) is linear in the entire studied concentration range. The sensitivity of the proposed analytical protocol defined as the slope of the calibration curve was equal to 0.17 A·M^−1^ for both, positive and negative signals. This value holds the expected order of magnitude and is similar to voltammetric detection sensitivities obtained for monocationinc molecules holding similar hydrodynamic dimensionality at macroscopic eLLI^8,48^. The lower limit of detection (LOD) for heroin was calculated using the equation:

1 $${\text{LOD }} = {3}.{\text{3SD}}/{\text{S,}}$$

whereas the lower limit of quantification (LOQ):

2 $${\text{LOQ }} = {1}0{\text{SD}}/{\text{S}},$$

where SD and S are the standard deviation of the intercept and the slope of the calibration curve, respectively (values taken from the calibrations curves linear fit equation).

The lower LODs for heroin dissolved in 10 mM HCl in 10 mM NaCl (pH = 2.0) calculated using Eq. (1) were equal to 1.3 µM for the positive and 1.9 µM for the negative signals. LOQs are correspondingly higher, this is 4.0 and 5.7 µM, respectively. These values are in line with the experimental concentration of heroin equal to 3.0 µM for which we have started observing quantifiable signal. Table 1 summarizes all physicochemical parameters of heroin and extracted from the voltammetric data. These include formal Galvani potential difference of ion transfer, formal Gibbs free energy of ion transfer, water – 1,2-dichloroethane partition coefficient, diffusion coefficient calculated from the Randles–Ševčík equation^49^ (see Fig. S1 with the corresponding discussion), forward to reversed peak current ratio, and forward to reversed peak to peak separation. Also, Table 1 contains the information used to prepare the ionic partition diagram shown as Fig. 1D (prepared based on CVs recorded in the presence of TPrA^+^ and heroin at different pH values as shown in Fig. S2). Here, the experimental points marked with black squares correspond to the Galvani half-wave potential difference extracted from the CVs recorded for different pH values. As the concentration of protons in the aqueous phase is altered we are also changing the concentration fraction of the monocationic and neutral forms of heroin. This affects its partitioning properties. According to our findings, at pH < 5, and for the ${\Delta }_{{\varvec{o}}{\varvec{r}}{\varvec{g}}}^{{\varvec{a}}{\varvec{q}}}{\varvec{\phi}}$ < ${\Delta }_{{\varvec{o}}{\varvec{r}}{\varvec{g}}}^{{\varvec{a}}{\varvec{q}}}{{\varvec{\phi}}}_{{\varvec{h}}{\varvec{e}}{\varvec{r}}{\varvec{o}}{\varvec{i}}{\varvec{n}}}^{\boldsymbol{^{\prime}}}$, heroin remains in the aqueous phase. When sufficient interfacial Galvani potential difference is applied from the external power source, this is at ${\Delta }_{{\varvec{o}}{\varvec{r}}{\varvec{g}}}^{{\varvec{a}}{\varvec{q}}}{\varvec{\phi}}$ > 82 mV, the cationic forms of heroin transfer from the aqueous to the organic phase following simple interfacial ion transfer reaction mechanism. This situation is marked with the red, solid line in Fig. 1D, for pH range up to 5, which is the best fit for the experimental points calculated using Eq. (3) ^29^:

3 $$\Delta {}_{org}{}^{aq}{\Phi }_{1/2}= \Delta {}_{org}{}^{aq}\Phi \mathrm{^{\prime}}+ \frac{RT}{F}\mathrm{ln}\left(\frac{{10}^{-pH}+{K}_{a}+{K}_{a}{K}_{D}}{{10}^{-pH}}\right),$$

where $${\Delta }_{org}^{aq}{\phi }^{^{\prime}}$$ is the formal ion transfer potential of heroin (calculated versus the $${\Delta }_{org}^{aq}{\phi }_{{TPrA}^{+}}^{^{\prime}}$$ = –0.091 V);^26^ pK*a*, *R, T, F* and *pH* hold their usual meaning, whereas *K*_*D*_ is the distribution constant defined as the ratio between neutral form of heroin in the aqueous ($${C}_{C}^{aq}$$) and the organic phase ($${C}_{C}^{org})$$:

**Table 1:** Summarized heroin physicochemical parameters.

Parameter	This work	Ref. data
pK_a_	–	7.6^50^
K_D_	550	–
ΔE_p_*/mV	73	–
D/µcm^2^·s^−1^	5.30	–
I^+^/I^−^*	1	–
${\Delta }_{org}^{aq}{\phi }^{^{\prime}}$/mV	82 ± 5	30^25^
*${\Delta }_{org}^{aq}{G}^{^{\prime}}$/kJ·mol^−1^	− 7.93*	− 3.20*
**$\mathrm{log}{P}_{DCE}$	− 1.4**	− 0.6**

*$\Delta {}_{org}{}^{aq}G\mathrm{^{\prime}}=- z \cdot F \cdot \Delta {}_{org}{}^{aq}{\Phi }^{\mathrm{^{\prime}}},$ where *z* and *F* is the charge and the Faraday constant, respectively.

**$\mathrm{log}{{P}^{\mathrm{^{\prime}}}}_\frac{aq}{org}= -\frac{z \cdot F \cdot \Delta {}_{org}{}^{aq}{\Phi }^{\mathrm{^{\prime}}} }{2.303 \cdot R \cdot T},$ where R (8.3145 $\frac{J}{mol\cdot K}$) is the gas constant and T is the temperature (288.15 K).

4 $${K}_{D}= \frac{{C}_{C}^{aq}}{{C}_{C}^{org}}.$$

The best fit for the experimental points depicted in Fig. 1D was obtained for *K*_*D*_ equal to 550. This means that heroin in the neutral form (pH > pK*a*) is relatively hydrophobic and predominantly partitions to the organic phase. At pH > 5, the mechanisms of the interfacial charge transfer reactions changes. The existence of the neutral form of heroin in the organic phase facilitates the transfer of protons from the aqueous to the organic phase upon forward polarization (from less to more positive potential). As the pH increases the fraction of the interfacially transferring charge originating from the simple heroin cation drops on account of facilitated transfer of protons.

**Figure 2:**
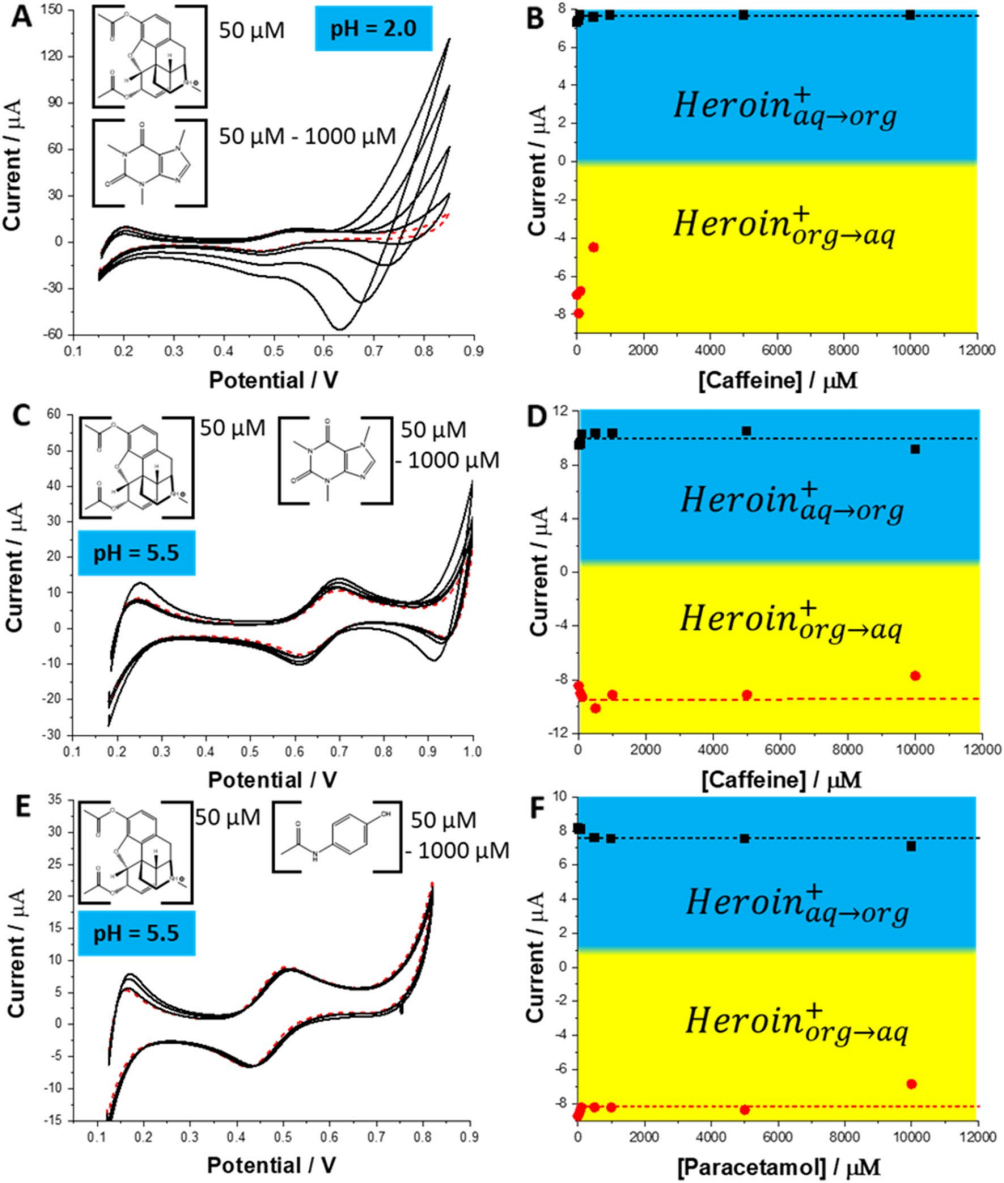
CVs recorded in the presence of [heroin] = 50 µM in the aqueous phase (dashed, red line) and (**A**) – [caffeine] = 50; 100; 500; 1000; 5000 and 10,000 µM at pH = 2.0; (**B**) – [caffeine] = 50; 100; 500; 1000; 5000 and 10,000 µM at pH = 5.5; and (**C**) – [paracetamol] = 50; 100; 500; 1000; 5000 and 10,000 µM at pH = 5.5. Scan rate was 20 mV s^−1^. Graphs (**B**), (**D**) and (**F**) correspond to the positive and negative ionic current originating from the heroin cation ion transfer (correlation between signal sign and direction of ion transfer is indicted on the figure) plotted in function of the cutting agent concentration, prepared based on CVs from section (**A**), (**C**) and (**E**), respectively.

To evaluate the performance of the eLLI based sensing protocol, the interfacial behavior of heroin was studied in the presence of its major cutting agents at the macroscopic soft junction. Figure 2 presents the CVs recorded in the presence of a fixed concentration of heroin equal to 50 µM and different concentrations of caffeine (Fig. 2A for pH = 2.0 and Fig. 2C for pH = 5.5) and paracetamol (Fig. 2E for pH = 5.5). The presence of either cutting agent in the analyzed sample in the concentration range from 50 to 10,000 µM (up to 10,000 times excess) only slightly affects the positive current reading. The difference between the positive peak current value extracted for 50 µM heroin in the absence of any cutting agent in the aqueous phase and the point displaying the highest discrepancy ([caffeine] = 10,000 µM, pH = 5.5) was 4.2% (see Fig. 2D). The overlay between negative ionic currents attributed to heroin and caffeine occurred at pH = 2.0. This is due to progressively shifting potential (towards less positive potential difference value) at which caffeine started transferring from the aqueous to the organic phase and further going back from the organic to the aqueous phase. Due to very intense caffeine back transfer (from the organic to the aqueous phase) signal proper analysis of the negative peak attributed to the heroin ion transfer is troublesome. This limitation can be improved by changing the pH of the aqueous to pH = 5.5 (see Fig. 2D,F). At such conditions neither caffeine nor paracetamol give a signal within the available potential window at any studied concentration. Also, this pH value still assures a 100% fraction of cationic heroine species present in the aqueous phase.

**Figure 3:**
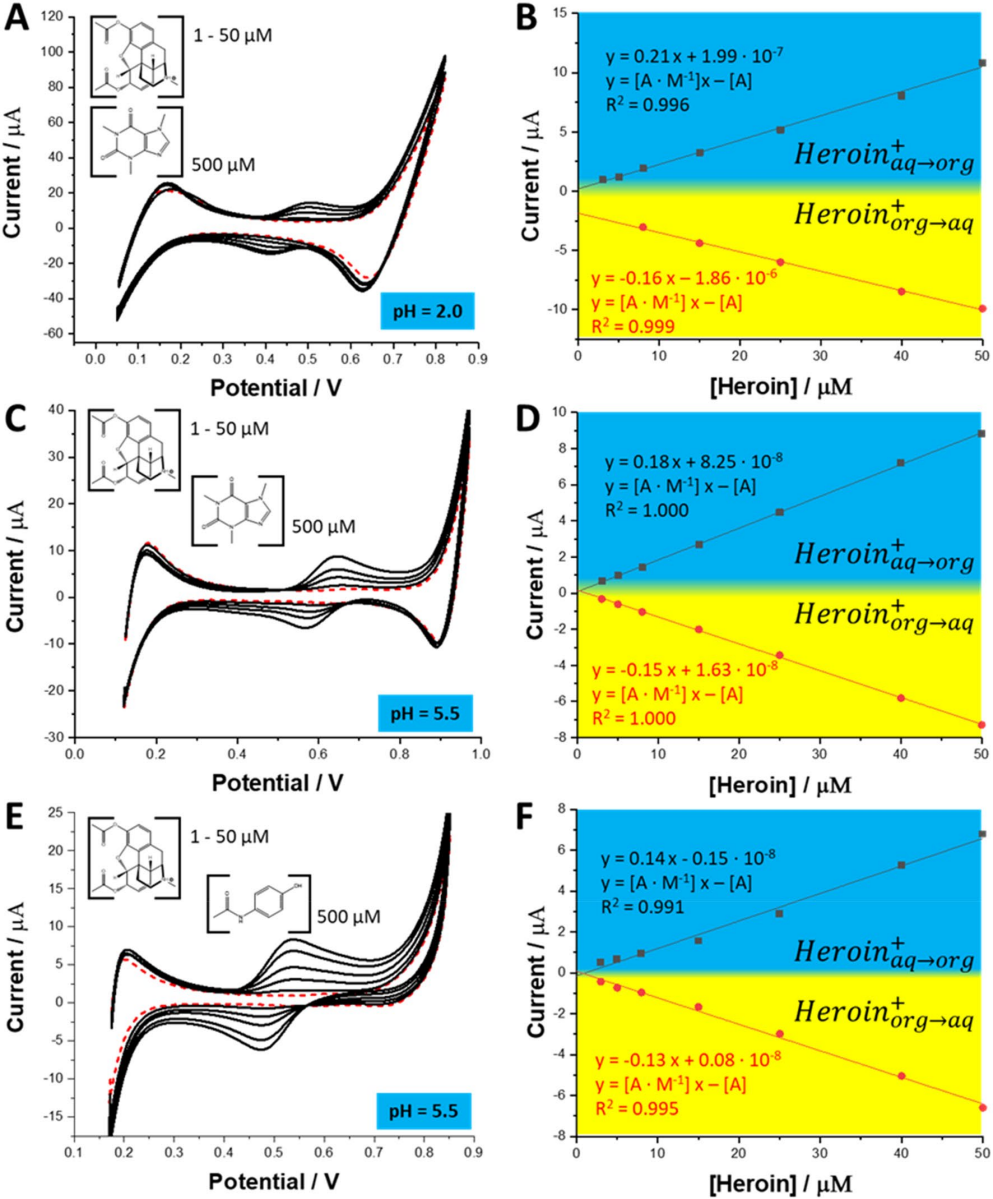
CVs recorded for the increasing concentration of heroin (from 1 to 50 µM) in the presence of (**A**) – 500 µM caffeine at pH = 2.0; (**C**) – 500 µM caffeine at pH = 5.5; and (**E**) – 500 µM paracetamol at pH = 5.5. The scan rate was 20 mV·s^−1^. (**B**), (**D**) and (**F**) are the calibration curves plotted based on the positive and negative peak currants prepared base on CVs from sections (**A**), (**C**) and (**E**), respectively.

Figure 3 shows the influence of the cutting agents added to the aqueous phase at the 10 times excess, studied at two pH values on the ionic currents recorded in the presence of heroin at different concentrations. In any case, we have found that the detection sensitivity (slope of the calibration curves) is very close to the value obtained for the blank reading. The voltammetric detection sensitivity plotted for the positive currents were found in the range from 0.14–0.21 A·M^−1^ (see Fig. 3B,D,F) which correlates well with 0.17 A·M^−1^ when only heroin and background electrolyte was present in the aqueous phase (see Fig. 1B). Similar findings are valid for the negative currents, as the voltammetric detection sensitivity was found in the range from − 0.13 to 0.16 A·M^−1^ being in line with − 0.17 A·M^−1^ for the reference measurement. With this in mind, we have concluded that the best sensing conditions are for the aqueous phase pH = 5.5 (lack of caffeine and paracetamol interference, 100% fraction of heroin cations assures high sensitivity).

**Figure 4:**
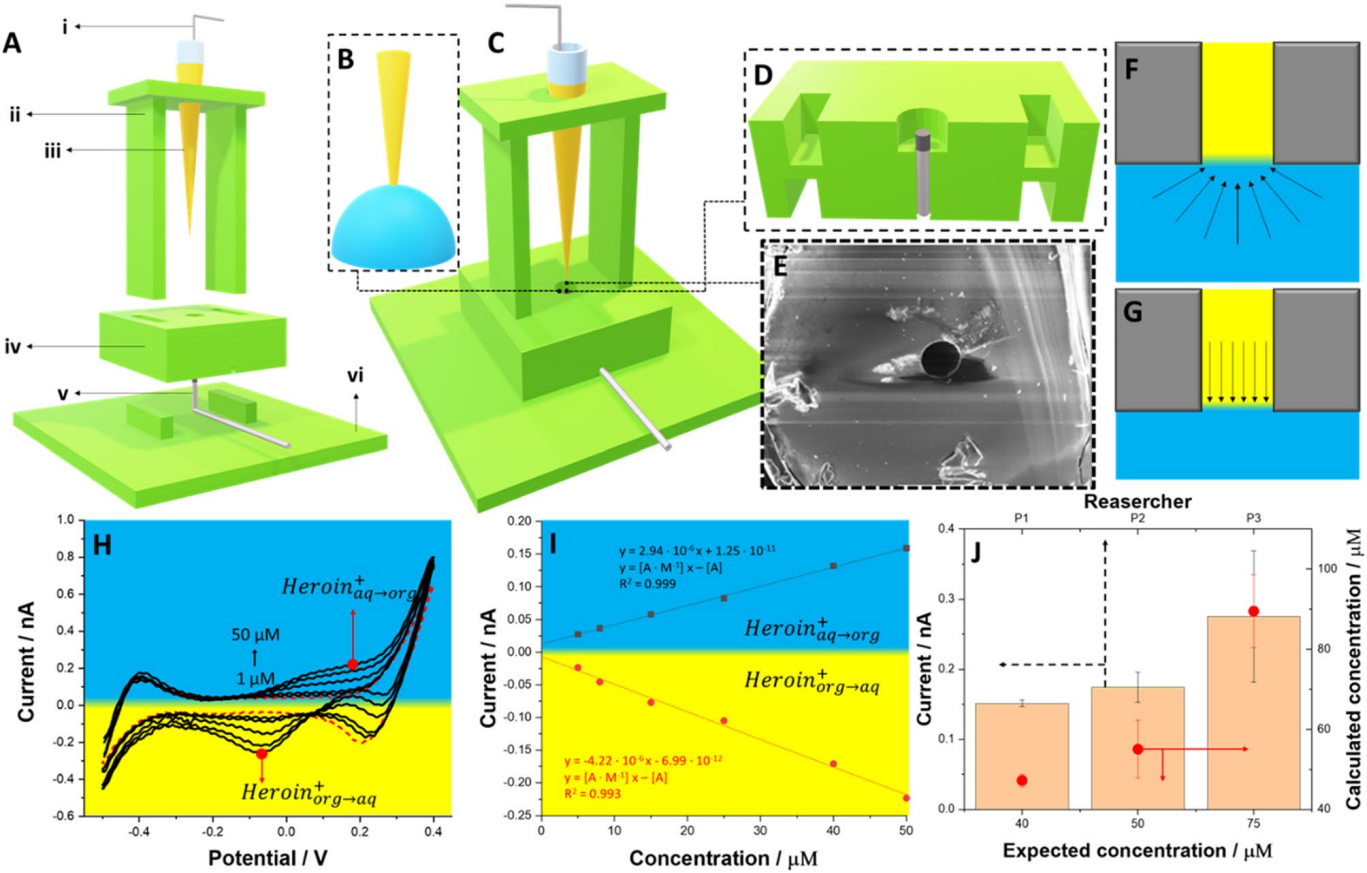
(**A**) – The sequential representation of the 3D printed platform used for the heroin detection in microliter volume droplet: (**i**) – is the Ag/AgTPBCl reference and counter electrode (in one) of the organic phase; (**ii**) is the support with pre-adjusted height hosting the tip filled with the (**iii**) organic phase; (**iv**) is the cell middle part with the opening for the droplet deposition; (**v**) is the Ag/AgCl electrode serving as the reference and counter electrode (in one) of the aqueous phase; (**vi**) is the printed platform support assuring entire cell stability. (**B**) – Zoom into the end of the tip filled with the organic phase immersed into the aqueous phase. (**C**) – Assembled platform. (**D**) – Cross section of the cell middle part (**iv**) showing the position of the (**v**) Ag/AgCl electrode. (**E**) – SEM micrography of the fused silica capillary pore having 25 µm diameter. (**F**) – Scheme showing the mass transfer from the aqueous to the organic phase governed by hemispherical diffusion. (**G**) – Scheme showing the mass transfer from the organic to the aqueous phase governed by linear diffusion. (**H**) – Series of CVs recorded at the microscopic LLI supported with fused silica capillary tubing for the increasing concentration of heroin (from 1 to 50 µM) in the presence of fixed [paracetamol] and [caffeine] = 500 µM (red dash line is CV recorded in the absence of heroin; pH = 5.5; scan rate = 20 mV·s^−1^). (**I**) – Calibration curves plotted based on the positive and negative peak currents deduced from part H. (**J**) – The chart showing the results of heroin detection from model formulations (bottom x, right y axes) together with repeatability test performed by three different persons (P1, P2, P3, upper x and left y axes). For axis meaning and details refer to the main text.

To reduce the amounts of consumed chemicals and needed sample volume which is subjected to the analysis we have developed and 3D printed modular platform that was further combined with the microscopic eLLI supported with the fused silica capillary tubing. Constructed platform arrangement is shown in Fig. 4A–D. 3D printing details together with the microscopic eLLI support fabrication details are described in the corresponding part of the electronic supporting information (see Sect. 4 in ESI). Briefly, the cell was composed of three main parts printed from polylactic acid: the saddle having 27 mm in height hosting the micropipette tip finished with a short section of the fused silica capillary tubing (see SEM micrography from Fig. 4E showing the top view of the pore with 25 µm in diameter); the middle part with a cavity placed in the centre being a compartment for the aqueous phase droplet with a volume up to 20 µL (the compartment was additionally equipped with the Ag/AgCl wire serving as the aqueous phase counter and the reference electrode); finally the bottom part assuring high stability. The microtip preparation protocol used as the eLLI support is published elsewhere^46^. The organic phase was always added to the microtip (volume ~ 5 µL) and the LLI was formed inside the aqueous phase droplet at the tip ingress (see Fig. 4B). Printing time of the entire cell takes up to 2 h whereas its estimated cost is lower than 1 euro (including electrodes).

Figure 4H shows a series of CVs recorded in the presence of heroin, caffeine and paracetamol at the miniaturized eLLI in the aqueous phase droplet using 3D printed platform. The expected shape of the obtained curves, sigmoidal signal for the heroin transfer from the aqueous to the organic phase, and peak like shaped response for the heroin transfer from the organic to the aqueous phase, is in line with the diffusion layer profiles established on the aqueous and the organic phase side of the LLI. The hydrophobic interior of the fused silica capillary (the interior of the pores surface of the commercially available capillaries is modified with methyl groups) assures wettability by only the organic phase, and hence, the pore is filled with this solution during the experiments. Mass transfer within the pore is governed by linear diffusion (see Fig. 4F) for which the corresponding voltammetirc signal is peak shaped. The capillary walls around the pore are made out of silica being preferentially wetted by the aqueous phase. As such, the mass transfer to the eLLI from the aqueous to the organic phase is governed by hemispherical diffusion layer profile giving sigmoidal voltammetric signals (see Fig. 4G). We have found that the detection of heroin in the presence of its major cutting agents (caffeine and paracetamol) can be successfully performed in the droplet as shown in Fig. 4I depicting the calibration curves plotted using the data from CVs shown in Fig. 4H. Obtained analytical parameters are summarized in Table 2. LOD for heroin detection under this conditions is equal to 1.7 µM being nearly the same as 1.3 µM for macroscopic system. With this in mind, first author of this work asked three different persons to prepare three fused silica capillaries based tips each and analyse given heroin formulation using a 3D printed platform.

**Table 2:** The summary of the electroanalytical parameters obtained for heroine alone and in the presence of caffeine and paracetamol for macroscopic and microscopic eLLI.

Parameter	Heroin alone/macroITIES	Heroin, paracetamol and caffeine/macroITIES	Heroin, paracetamol and caffeine/µITIES
Sensitivity ( +)/A·M^−1^	0.17	0.17	2.94 · 10^−6^
Sensitivity ( −)/A·M^−1^	− 0.17	− 0.14	− 4.22 · 10^−6^
LOD ( +)/µM	1.35	1.33	1.67
LOD (−)/µM	1.91	2.03	3.97
LOQ ( +)/µM	4.05	3.99	5.01
LOQ (−)/µM	5.74	6.10	11.91

The result of this verification study is summarized in Fig. 4J which correlates (i) expected concentration of heroin in the analyzed sample with the concentration found by person performing analysis (see red circles attributed to the bottom x – right y axes), and (ii) the experimental reproducibility deduced from the obtained current for three constructed tips used for the heroin analysis (columns, upper x – left y axes). Figure 4J was prepared using CVs recorded in the 3D printed platforms at miniaturized eLLI – for examples please refer to Fig. S3 from electronic supporting information. The recovery values by P1, P2 and P3 were found to be 117.5%, 110% and 119%, respectively (the average of three independent experiments) which is in line with analytical standards. Slightly elevated values can originate from the difficulty related to positive current read-out (sigmoidal wave). The repeatability of the experimental data by P1 and P2 provided the smallest error bars (for P1 the average current for the given heroin concertation for the forward scan was 0.15 ± 0.01 nA whereas for P2 this was equal to 0.17 ± 0.02 nA) indicating high precision. P3 obtained a data set with slightly higher error bar (0.27 ± 0.10 nA) still being in line with analytical standards. All these findings indicate that 3D printed platforms hosting a microliter volume of the aqueous phase, together with miniaturized eLLI can be successfully used for the heroin detection in the presence of caffeine and paracetamol which are electrochemically active at conventional solid electrodes^23^. One of our limitations, the volatility of the organic phase, can be overcome by backfilling the microtip with the aqueous phase or covering it with a foil (e.g. made out of parafilm) that will prevent the organic phase evaporation. This is further confirmed by the comparison of the electroanalytical output of the proposed methodology with the existing state-of-the-art summarized in Table 3. With the LOD value having the same order of magnitude as compared with electroanalytical protocols provided using screen-printed electrodes, and lack of interference originating from the paracetamol and caffeine (both cutting agents are active at the carbon-based electrode), we offer a functional sensor that can be successfully applied to detect heroin directly from street samples. For the latter, the prior dissolution of the street powders in 10 mM HCl e.g. performed in the Eppendorf tube, is a must.

**Table 3:** Comparison of the electroanalytical parameters for the presumptive electrochemical heroin detection.

Transducing element	Sensitivity/A·M^−1^	LOD/µM	References
Preanodized graphite screen printed electrode	0.019	5.2	^51^
Screen printed electrode	0.006	3.3	^52^
Ru(bpy)_3_^2+^* immobilized in zeolite Y modified carbon paste electrode	–	1.1	^45^
Miniaturized liquid–liquid interface	0.17	1.3	This work

*Ru(bpy)_3_^2+ ^— tris(2,29-bipyridyl)ruthenium(II).

## Conclusions

In this work, we have studied the interfacial behaviour of heroin alone and in the presence of caffeine or/and paracetamol at the electrified liquid–liquid interface. Two latter compounds are the main cutting agents found in the vast majority of heroin street samples. Heroin undergoes the electrochemically controlled ion transfer across the water – 1,2-dichloroethane solutions interface within the available potential window. At optimized aqueous phase pH equal to 5.5 the cutting agents either affected the voltammetric signal originating from the heroin interfacial transfer nor the voltammetric detection sensitivity. To fully reveal the analytical potential of the developed procedure we have 3D printed the sensing platform hosting up to 20 µL of the aqueous phase and the end of the microtip (fused silica capillary having 25 µm as the internal pore diameter) being used as the support of the polarizable soft junction. We have proved that the developed heroin sensing platforms fully meet forensic demands.

## Supplementary Information

Supplementary Figures.

## Data Availability

The datasets used and/or analysed during the current study available from the corresponding author on reasonable request.
