# Supplementary material for: Heroin detection in a droplet hosted in a 3D printed support at the miniaturized electrified liquid-liquid interface
Source: Sci Rep. 2022 Nov 3;12:18615. doi: 10.1038/s41598-022-21689-0 (PMC9633610; doi:10.1038/s41598-022-21689-0)
Supplement: Supplementary file 1 — Supplementary Figures. [file 41598_2022_21689_MOESM1_ESM.docx]

**Electronic Supporting Information**

**Heroin detection in a droplet hosted in a 3D printed support at the miniaturized electrified liquid-liquid interface**

Paulina Borgul,^a^ Karolina Sobczak,^a^ Karolina Sipa,^a^ Konrad Rudnicki,^a^ Slawomira Skrzypek,^a^ Anna Trynda,^b^ Lukasz Poltorak*^a^

a. Department of Inorganic and Analytical Chemistry, Electroanalysis and Electrochemistry Group, Faculty of Chemistry, University of Lodz, Tamka 12, 91-403 Lodz, Poland.

b. Chemistry Department, Central Forensic Laboratory of the Police, Al. Ujazdowskie 7, 00-583 Warsaw, Poland.

*Corresponding author: [lukasz.poltorak@chemia.uni.lodz.pl](mailto:lukasz.poltorak@chemia.uni.lodz.pl)

**Table of content:**

Figure S1. Scan rate dependency for heroin…………………………………………….…………………page S2

Figure S2. Additional ITVs. The effect of pH………………………………………………………………..page S2

Figure S3. ITVs recorded with miniaturized ITIES………………………………………………………..page S3


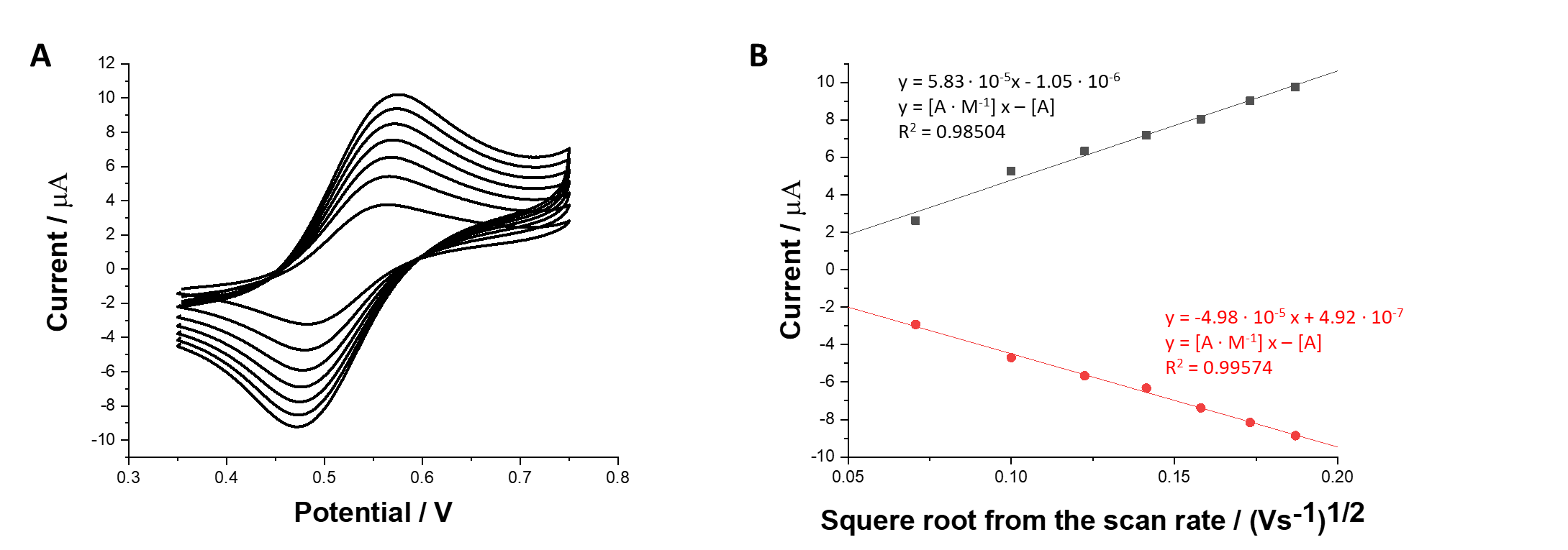


**Figure S1.** **A** – Scan rate dependency for the 50 µM heroin recorded for increasing scan rates: 5; 10; 15; 20; 25; 30 and 35 mV s^-1^. The aqueous phase had pH of 2. **B** – The corresponding current curves show forward (positive) and reversed (negative) currents plotted in function of the scan rate.

**
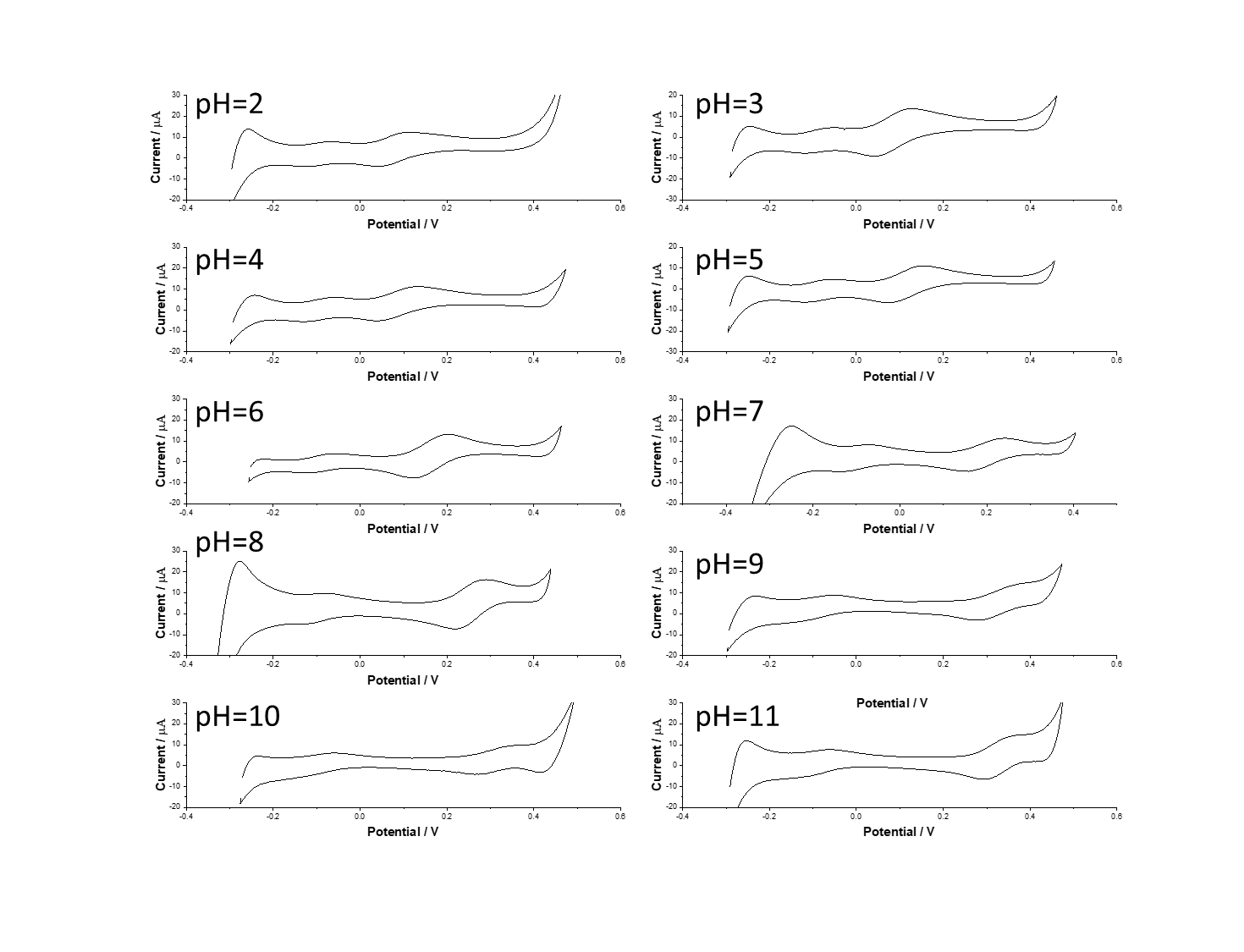
**

**Figure S2.** The series of ITVs recorded at different pH values (2-11) for heroin (50 µM) in the presence of TPrA+ (model ion - tetrapropylammonium cation, 25 µM). Scan rate was
20 mV s^-1^. The aqueous phase was the BRB solutions.


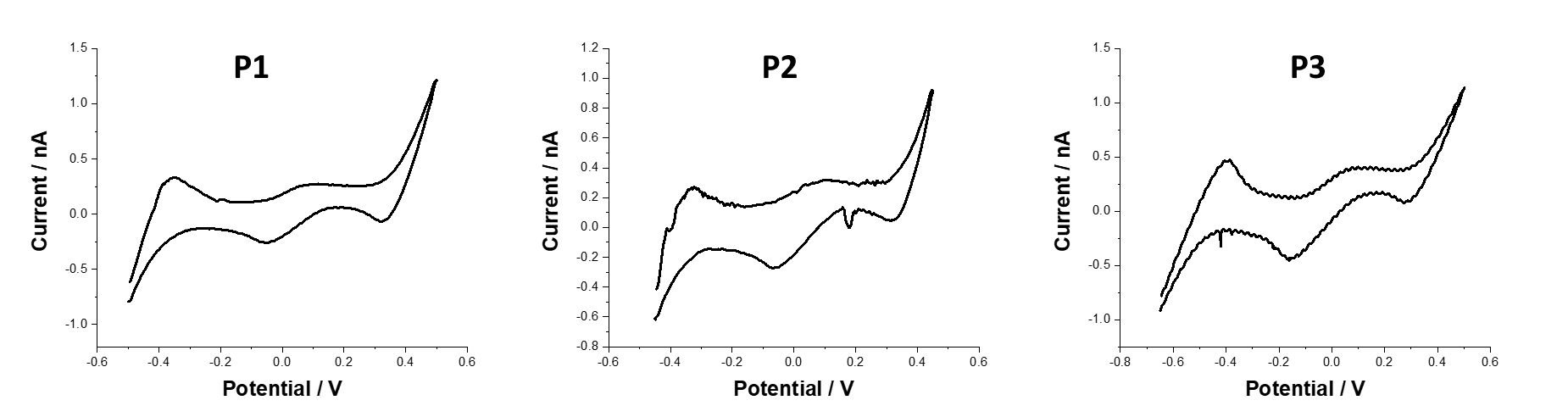


**Figure S3.** ITVs recorded for individual people participating in the repeatability and reproducibility test. The theoretical heroin concentrations were 40 µM, 50 µM and 75 µM for P1, P2 and P3, respectively The scan rate was 20 mV s^-1^.
